# Supplementary material for: Health care workers’ self-perceived infection risk and COVID-19 vaccine uptake: A mixed methods study
Source: PLOS Glob Public Health. 2023 Jun 7;3(6):e0001223. doi: 10.1371/journal.pgph.0001223 (PMC10246783; doi:10.1371/journal.pgph.0001223)
Supplement: Attachment — Submitted filename: PLOS_Response to Reviewer_Resubmission.docx [file pgph.0001223.s001.docx]

**Health care workers’ self-perceived infection risk and COVID-19 vaccine uptake (PLOS One Submission)**

**Response to reviewer’s matrix:**

| **SN** | **EDITOR’S COMMENTS** | **AUTHOR’S RESPONSES** |
| --- | --- | --- |
|  |  |  |
|  | **Introduction:** the authors should better contextualize the study by addressing the availability of a national policy / guideline on vaccination of health care workers against preventable diseases (e.g., hepatitis B, influenza) including COVID-19.  Also provide information on the current COVID-19 vaccine coverage rates within the general population, and among relevant populations groups like health care workers (globally versus regionally versus locally in Tanzania). | Thank you for this suggestion.  No national guideline was retrievable on vaccination of health workers in Tanzania. Available guidelines on HCW vaccination appear to be disease specific.  However, information on the recommendations from the National COVID-19 committee regarding HCWs vaccinations has been provided. |
|  | **Methodology:** The authors should provide a justification for choosing thematic analysis as the most appropriate method for the data collected. | The editor’s observation is well noted and appreciated.  A justification for choosing thematic analysis is provided, and was applied to facilitate immersing into the data to methodically and thoroughly identify themes and patterns to aid an in depth understanding of participants’ opinions, experiences, and risk perceptions towards COVID-19 across the dataset |
|  | **Results:** a full outline / list of the core emergent themes should be explicitly reported in the Results section. | Three emergent themes were identified from the analysis: 1. perceived risk of being infected; 2. risk of infecting others and 3. perceived reduced risk to infection due to use of COVID-19 vaccines.  The emergent themes have now been reported in the Results section. |
|  | **Discussion:** The authors should fully appraise the findings of this study against similar studies conducted in Africa and elsewhere (providing specific examples where relevant), exploring health care workers’ perceived risk of COVID-19 and uptake of COVID-19 vaccination. | A more extensive appraisal of current findings against available relevant literature has been conducted.  Several papers have been added in the discussion related to similar papers in Africa and from recommendations provided by the reviewers. |
|  | **Limitations** the authors should address issues around recall bias and the limitation of not using health care workers’ electronic COVID-19 vaccination records/certificates to confirm vaccination history.  It is also unclear to me if health care workers were asked to provide reasons for why they were not vaccinated. Is there a reason the authors chose not to collect such data. | Limitations including recall and interviewer bias have been acknowledged and discussed.  The HCWs were not requested to provide reasons for not being vaccinated. However, another research team conducted thorough inquiries on the HCWs perceptions on COVID-19 vaccines. This information will be presented in a separate study. |
|  | **GRAMMATICAL AND OTHER ERRORS:**  1. The authors should take note of editorial and grammatical errors throughout the document and ensure that these are resolved when revising the manuscript. Examples include but are not limited to the following:   - Line 79 pg 8; “…first nation-wise COVID 19 vaccinations…” Do the authors mean “nation-wide”? - Inconsistency in writing COVID-19 and SARS-CoV-2 - Lines 97 – 99 pg 9; “While being a high-risk group…associated positive protective behaviors” This sentence is unclear. Kindly rephrase for better clarity. - Line 235 pg 17; “The higher perceived risk to COVID 19 infection perceptions…” Please rephrase this sentence for ease of clarity. - Line 325 pg 21; “However, consisted availability of appropriate and required PPEs…” Please rephrase this sentence for ease of clarity. - Lines 375 – 377 pg 24; “Targeted information the public on the beneficial role…” Please rephrase for clarity.   2. Lines 82 – 83 pg 8; “A total of 2,431,769 vaccine doses administered by the end of year 2021 under COVAX facility” this sentence appears to be hanging or incomplete. No date as to when this tally was reach has been supplied.  3. Lines 83 – 85 pg 8; “While vaccine availability may not have been a challenging option initially…” This statement may be misleading. The challenges in availability of, and access to COVID-19 vaccines across the African region at the height of the pandemic have been widely acknowledged. Further to this, the negative far-reaching consequences of withholding vaccines from the region on vaccine acceptance and uptake at the population level may be immeasurable but existent. Please consider rephrasing this statement.  4. The sample size in the Abstract section is 1386 while that in the results section is 1368. Please resolve this disparity. In addition, kindly address if all health care workers invited agreed to participate in this study. In other words, what are the recruitment versus enrolment numbers for this study.  5. While Table 3 is presented, it is not cited within the text. Kindly make reference to Table 3 within the reference section for ease of clarity.  6. Lines 336 – 337 pg 22; “It has been widely reported that high perceived risk of contracting COVID 19 is a significant predictor of vaccine acceptance.” Please provide reliable references to support this statement.  7. Line 340 – 342 pg 22; “One probable explanation for this…people in Africa consider the vaccines as unnecessary…” Given that this was a single country, multi-site study, is this inference and generalization founded?  8. Line 344 – 347 pg 22; “However, this study has shown…especially vaccine safety concerns…” did the authors report on vaccine safety concerns among the study population?  9. I was unable to access the supplementary file “Data review URC” using the link provided. | **Drs. Harrieth and Belinda**  1. Thank you for the comments and the specifically pointed statements. We have corrected all the grammatical errors and rephrased some statements as suggested.  2. Thank you for the observation. The line has been revised and currently reads as:  “In Tanzania, a total of 39,392,419 vaccine doses were administered by the 22nd of March 2023 under COVAX facility”  3. We thank the reviewer for this comment. We have rephrased this statement to only infer to our setting as this may not be very generalizable. It now reads as:  “Currently, access to COVID-19 vaccine in Africa has improved; however, vaccines acceptance and hesitancy influenced by social, political, and religious factors may contribute to low vaccine uptake”  4. Thank you for the comment regarding sample size discrepancies. It was a typographical error.  We have now corrected the sample size in the abstract section to match the correct sample size from the results (1368).  Regarding response rates: A small number of the targeted HCWs were not present at their places of work during the current survey. However, all the HCWs that were approached consented to participate in the study.  5. Thank you for the comment, we have now cited Table 3 in the text.  6. Thank you for the comment, several references have now been added.  7. Thank you for the comment.  The statement has been reworded to show the tentative nature of the assertion more clearly. It now reads as:  “One probable explanation for this could be that a significant proportion of people in Africa including Tanzania, consider the vaccines as unnecessary, and that alternatives to COVID-19 vaccination exist”.  8. The current study did not report on vaccine safety concerns, the statement has been rephrased and currently reads as:  “Other factors, especially vaccine safety concerns have been shown to outweigh the perceived disease risks in determining vaccine acceptance as indicated in a systematic review among healthcare workers in Africa”  9. Please find attached the link to the raw dataset from this study:  <https://datadryad.org/stash/share/6OGisFQKD4MQ87HCBkHzhx6iflTT7dDHCfHk5dryYmk> |
|  | **REVIEWER 1 COMMENTS** |  |
|  | **Results:** I would like to recommend if they consider using Univariate binary logistic regression to estimate the magnitude of the statistical associations and perform multivariate logistic regression to identify the predictors of SARS-CoV-2 vaccine uptake and high-risk perception. In addition to controlling for potential confounders. | Thank you very much for these recommendations.  Table 2 has now been transformed into a univariate binary logistic regression model showing the relationship between COVID-19 risk perception and vaccination status.  Multivariate logistic regression was not performed in an effort to rein in the extent of our results. |
|  | **Results:** If vaccination dates are available analyzing risk perception per pandemic situation period would be valuable as well. | Thank you for this suggestion.  Unfortunately, the vaccination dates are unavailable to the research team. |
|  | **Discussion:** In the discussion part I would recommend mentioning changes in pandemic situation between November 2021 and January 2022 which could influence COVID-19 risk perception among HCWs. | Thank you for this suggestion.  However, due to the cross-sectional nature of the study, assessment of pandemic factors that may influence risk perceptions among HCW could not be made. This has now been included as a limitation in the discussion, explaining the evolving nature of the findings, and the inability of our study to predict or project change. |
|  |  |  |
|  | **REVIEWER 2 COMMENTS** |  |
|  | **GENERAL COMMENTS:** The manuscript does not read well in some sections with many instances of poor grammar. I strongly recommend that you make use of a language editor.  The abstract contains abbreviations/acronyms which have not been previously defined.  The authors mix spellings of American English and UK English. I would suggest they stick to one style throughout | We thank the reviewer for this comment, we have thoroughly revised the manuscript to correct grammatical errors.    All abbreviations are now defined as they first appear in the manuscript.  American English has been used throughout the manuscript. |
|  | **Background:**  1. The data of confirmed COVID-19 cases and deaths is very dated. The same for number of vaccine doses administered under the COVAX facility. Please update with latest data  2. Acceptability, and willingness of the population to vaccinate is tautology  3. Ensure consistency in terminology; COVID-19 versus COVID 19. SARS-CoV-2  4. Line 79, nation-wide instead of nation-wise  5. Please add a reference to the bold statement in lines 83 – 85.  6. Add quotation marks to the definition of vaccine hesitancy (lines 104 -105). | 1. We have updated the Data on COVID-19 cases, deaths and vaccines administered to that of the 22^nd^ March 2023.  2. Thank you for the comments, we have struck out “willingness” from the statement.  3. We have now consistently used COVID-19 in the manuscript.  corrected all the grammatical errors  4. Thank you for the observation, we have enacted the change as advised.    5. References have now been added  6. Quotation marks have been added to the definition of vaccine hesitancy |
|  | **Methodology:**  1. Study sites – It is not clear the reason why Dar es salaam was purposefully chosen?  2. Sample size determination – what is the population from which the sample size determination (line 141-143) was determined?  3. How were HCWs chosen for participation in the study? Were all available HCWs invited to participate and interviews carried out until the minimum sample size was reached? Or were HCWs randomized for participation in the study? This is not clear.  4. HCWs was defined earlier in the manuscript. No need to use the full term thereafter.  5. Please add details about the pilot test.  6. Is there any special reason why the qualitative component of the study was not carried out in all seven regions? Why the choice of four regions instead of all regions (or the one cosmopolitan (Dar es salaam) region)?  7. Is ‘hospital in charges’ (line 157) an official designation or title in the Tanzanian health system?  8. More details about the questionnaire. What did the validated tool contain? Likert scale to measure risk perception?  9. Line 170 - ‘Consent information was administered?’ Do you mean the study information was given and consent obtained?  10. Line 175, please define the abbreviations, IDI and FDG in full.  11. It is not clear how the authors determined high risk perception and low risk perception. What was the cut-off point at which a participant can be regarded as having a high risk perception to COVID-19? | 1. Information on reason for choosing Dar es Salaam has now been provided as follows:  “Dar es salaam was purposively selected to be included because of it is the largest city in Tanzania, and it is the major port of entry to the country from international arrivals. The researchers believed that it is important to capture the HCW population perceptions from a cosmopolitan city.”  2. Information on sample size determination has now been provided as follows:  “Study population from which the sample size was determined included all cadres of HCWs at all levels of healthcare service provision in Tanzania. This included any medical, dental, laboratory, pharmaceutical or nursing staff working in a medical institution as officially registered through their professional associations.”  3. Information on how HCWs were chosen to participate in the study has been provided as follows:  “… all available HCWs were invited to participate, and interviews carried out until the minimum sample size was reached.”  4. Thank you for the observation, the abbreviation HCW has been used.  5. Details about pilot testing have now been provided as follows:  “The pilot testing of the study tools was done through a randomly selected group of healthcare professionals from Muhimbili University of Health and Allied Sciences, to whom the link to the ODK tool was shared for testing. Feedback on the shortcomings of the tools was incorporated in the revised final version. “  6. The reason why the qualitative component was only carried out in selected regions has now been provided as:  “The qualitative component of the study was conducted in four of the seven regions where the quantitative study took place due to resource limitations.”  7. Yes, “health facility in-charge” is an official designation in Tanzanian health system.  8. More details of the questionnaire provided as follows:  “The questionnaire contained both closed and open-ended questions for assessment of awareness and knowledge components. Risk perception towards COVID-19 infection was measured on Likert scale.”  9. Thank you for this observation. Rephrasing of the statement was done and it now reads as:  “Then, they provided study information to the HCWs and obtained informed consent in a quiet, private place around the health facility.”  10. The abbreviations IDI (In-depth interview) and FGD (Focus group discussions) have been provided in full.  11. The cut-off point of risk perception was determined from the provided response in the Likert scale. Additional details on the dichotomization of the responses have now been added to the manuscript as:  “ … the options “not at all, very low risk, low risk, and medium risk” were categorized into “Low risk” and options “high risk and very high risk” into “High risk” for dichotomization of the provided responses.” |
|  | **Results (Quantitative)**  Please state the response rate of the participants. 1. RESULTS – Table 1, Intervals for the work experience (<6 and 6+) are not logical.  2. Table 2 results should be shown as Odds Ratio. This would be a more a useful result.  3. Please use the same number of decimal places for your percentages | 1. Thank you for this comment.  Due to high variation in work experience, it was dichotomized at the median for analysis purposes. This information has now been included in the manuscript as:  “…. whereas work experience was dichotomized at its median (<6 years, 6+ years).”  2. Thank you for this suggestion. Table 2 has been transformed into a univariate binary logistic regression model showing the relationship between COVID-19 risk perception and vaccination status.  3. The same number of decimal places has been maintained in Table 2. |
|  | **Results (Qualitative)**  More probing of participants of the qualitative arm of the study in order to document their risk behaviours, experiences, and even recommendations on how to improve the vaccine uptake and/or risk perception | Thank you for this suggestion.  During thematic analysis, three main themes emerged: 1. perceived risk of being infected; 2. risk of infecting others and 3. perceived reduced risk to infection due to use of COVID-19 vaccines.  These are the themes around which the current qualitative findings are clustered around.  However, in subsequent research undertakings we will strive to capture more information from various specified aspects. |
|  | **Discussion:**  The Discussion is very thin. The authors missed out on an opportunity to discuss their findings and compare with many other studies carried out in Tanzania  https://www.mdpi.com/2076-393X/10/9/1429; https://iariw.org/wp-content/uploads/2022/10/Masele_Daud_IARIW-TNBS-2022-1.pdf  and other international studies such as  (<https://journals.plos.org/plosone/article?id=10.1371/journal.pone.0250017>;  <https://www.frontiersin.org/articles/10.3389/fpsyg.2020.02166/full>  <https://journals.plos.org/plosone/article?id=10.1371/journal.pone.0242471>  <https://www.tandfonline.com/doi/full/10.2147/RMHP.S310289>;  <https://intjem.biomedcentral.com/articles/10.1186/s12245-021-00341-0>;  <https://www.sciencedirect.com/science/article/pii/S0195670120302784?casa_token=OvEn9vM2IzQAAAAA:umHRhFRZiScbi163sbEXlt0r6gV1ZFMAJcPpsPu979DDzqi4nhdqqjwCSH1TOEleXf2kanJp4wPg>  to mention a few studies | Thank you very much for this comment and your provision of other relevant literature for our reference.  A more extensive appraisal of current findings against available relevant literature has now been conducted. Several papers have been added in the discussion related to similar papers in Africa and from recommendations provided by the reviewers. |
|  | **Recommendations and Policy Implications:**  Clear recommendations should be provided based on the findings of the study.  The statements under the ‘Policy implications’ section of the manuscript are too general. The authors should state how future/current policy for vaccination and risk behaviours can be influenced and by the findings of the study | Thank you for the comment.  The Policy Implications section has now been made more explicit and recommendations based on our findings. |
|  | **References:**  Reference #3 and #6 needs to be fixed (start with the author).  Add access dates to all online references | **Drs. Harrieth and Belinda**  The references have been modified with authors names at the beginning, access dates are added to all online references. |
