# Supplementary material for: Health care workers’ self-perceived infection risk and COVID-19 vaccine uptake: A mixed methods study
Source: PLOS Glob Public Health. 2023 Jun 7;3(6):e0001223. doi: 10.1371/journal.pgph.0001223 (PMC10246783; doi:10.1371/journal.pgph.0001223)
Supplement: Attachment — Submitted filename: PLOS_Response to Reviewers_Resub 2.docx [file pgph.0001223.s002.docx]

**Health care workers’ self-perceived infection risk and COVID-19 vaccine uptake (PLOS One Submission)**

**Response to reviewer’s matrix: Second Review**

| **SN** | **EDITOR’S COMMENTS** | **AUTHOR’S RESPONSES** |
| --- | --- | --- |
|  |  |  |
|  | Please review your reference list to ensure that it is complete and correct. If you have cited papers that have been retracted, please include the rationale for doing so in the manuscript text, or remove these references and replace them with relevant current references. Any changes to the reference list should be mentioned in the rebuttal letter that accompanies your revised manuscript. If you need to cite a retracted article, indicate the article’s retracted status in the References list and also include a citation and full reference for the retraction notice. | We thank the editor for the comments, all references have been reviewed and necessary changes made. We have not included any retracted article.  Reference 1-Replaced with a more relevant reference  Reference 2-Replaced with an updated reference  Reference 3-Replaced with an updated reference  Reference 5-Updated with a correct format  Reference 6-Reformatted  Reference 7-Reformatted  Reference 8-Reformatted  Reference 9-Format updated  Reference 11-Reformatted |
|  | **REVIEWER 2 COMMENTS** |  |
| 1. | Line 3 of the abstract; ‘healthcare’ is different for that used in the title ‘health care’. Also check Lines 80, 143, 244, 299. | Thank you for the comment. We have made necessary changes and replaced health care workers with the corresponding abbreviations thereafter. |
| 2. | HCWs is defined in line 80. No need to use the unabbreviated text thereafter. | Thank you for this suggestion. We have made the required revisions and ensured subsequent usage of the abbreviated term “HCW” |
| 3. | Are the terms healthcare workers, healthcare professionals, healthcare personnel interchangeable? I recommend using health care workers throughout the manuscript. | Thank you for this observation and recommendation. We have adopted health care workers (HCWs) throughout the manuscript. |
| 4. | Line 68-69. The data of confirmed global COVID-19 cases and deaths is very dated. Please update with latest data | Thank you for the comment.  We have updated the numbers based on the current WHO coronavirus dashboard and made corresponding-recitations. |
| 5. | Line 192, FGD already defined earlier. | Thank you for this comment. The term FGD has now been used instead of its long form.  We have replaced Focused Group Discussion with FDG after line 181 |
| 6. | Line 265, change COVID 19 to COVID-19 | Thank you for this suggestion. We have changed COVID 19 to COVID-19 in the mentioned line and ensured the same throughout the manuscript |
| 7. | Line 299, HCWs instead of health care workers. | Thank you for this suggestion. We have replaced health care workers with HCWs. |
| 8. | Table 2 – please double check the 95% CI values. Does not seem correct | Thank you for this observation. It was a typographical error.  The 95%CI values for the 1.535 OR are now correctly written as (1.197, 1.967). |
| 9. | Reference #2 is incomplete. | The reference has been updated and currently reads as;  World Health Organization(WHO), “Coronavirus disease (COVID-19) Dashboard,” 2023. https://covid19.who.int/ (accessed Apr. 27, 2023). |
| 10. | The authors in their response claim the following ‘A more extensive appraisal of current findings against available relevant literature has now been conducted. Several papers have been added in the related to similar papers in Africa and from recommendations provided by the reviewers.’ This is not reflected in the discussion with the same references used as in the original submission. | Thank you for the comment. The references below with corresponding text were added in the discussion. These were not in the originally submitted manuscript.  **28-** İ. Dolu, Z. Turhan, and H. Yalnlz Dilcen, “COVID-19 Vaccine Acceptance is associated with Vaccine Hesitancy, Perceived Risk and Previous Vaccination Experiences,” *Disaster Med. Public Health Prep.*, vol. 17, Dec. 2023, doi: 10.1017/DMP.2021.370.  **29-** A. AlShurman, A. Fozia Khan, C. Mac, M. Majeed, and Z. Ahmad Butt, “What demographic, social, and contextual factors influence the intention to use COVID-19 vaccines: a scoping review,” *mdpi.com*, 2021, doi: 10.3390/ijerph18179342.  **30-** N. Patterson, V. Paz-Soldan, R. Oberhelman, L. Moses, A. Madkour, and T. Miles, “Exploring perceived risk for COVID-19 and its role in protective behavior and COVID-19 vaccine hesitancy: a qualitative study after the first wave,” *BMC Public Health*, vol. 22, Mar. 2022, doi: 10.1186/s12889-022-12900-y.  **32-** C. Moucheraud *et al.*, “Uptake of the COVID-19 vaccine among healthcare workers in Malawi,” *Int. Health*, vol. 15, no. 1, pp. 77–84, 2023, doi: 10.1093/inthealth/ihac007.  **33-** D. Yilma *et al.*, “COVID-19 vaccine acceptability among healthcare workers in Ethiopia: Do we practice what we preach?,” *Trop. Med. Int. Heal.*, vol. 27, no. 4, pp. 418–425, Apr. 2022, doi: 10.1111/tmi.13742.  **35-** E. Konje *et al.*, “The Coverage and Acceptance Spectrum of COVID-19 Vaccines among Healthcare Professionals in Western Tanzania: What Can We Learn from This,” *mdpi.com*, 2022, doi: 10.3390/vaccines10091429. |
